# High yielding biomass genotypes of willow (*Salix* spp.) show differences in below ground biomass allocation

**DOI:** 10.1016/j.biombioe.2015.04.020

**Published:** 2015-09

**Authors:** Jennifer Cunniff, Sarah J. Purdy, Tim J.P. Barraclough, March Castle, Anne L. Maddison, Laurence E. Jones, Ian F. Shield, Andrew S. Gregory, Angela Karp

**Affiliations:** a Agroecology Department, Rothamsted Research, Harpenden, Hertfordshire, AL5 2JQ, UK; b Institute of Biological, Environmental and Rural Sciences (IBERS), Aberystwyth University, Gogerddan, Aberystwyth, Ceredigion, SY23 3EE, UK; c Sustainable Soils and Grassland Systems Department, Rothamsted Research, Harpenden, Hertfordshire, AL5 2JQ, UK

**Keywords:** Willow, Biomass, Genotypes, Roots, Allocation, Carbon accumulation

## Abstract

Willows (*Salix* spp.) grown as short rotation coppice (SRC) are viewed as a sustainable source of biomass with a positive greenhouse gas (GHG) balance due to their potential to fix and accumulate carbon (C) below ground. However, exploiting this potential has been limited by the paucity of data available on below ground biomass allocation and the extent to which it varies between genotypes. Furthermore, it is likely that allocation can be altered considerably by environment. To investigate the role of genotype and environment on allocation, four willow genotypes were grown at two replicated field sites in southeast England and west Wales, UK. Above and below ground biomass was intensively measured over two two-year rotations. Significant genotypic differences in biomass allocation were identified, with below ground allocation differing by up to 10% between genotypes. Importantly, the genotype with the highest below ground biomass also had the highest above ground yield. Furthermore, leaf area was found to be a good predictor of below ground biomass. Growth environment significantly impacted allocation; the willow genotypes grown in west Wales had up to 94% more biomass below ground by the end of the second rotation. A single investigation into fine roots showed the same pattern with double the volume of fine roots present. This greater below ground allocation may be attributed primarily to higher wind speeds, plus differences in humidity and soil characteristics. These results demonstrate that the capacity exists to breed plants with both high yields and high potential for C accumulation.

## Highlights

• SRC willows are a source of biomass and act as carbon (C) sinks.
• Biomass allocation was measured in 4 willow genotypes grown in two UK field sites.
• The greatest yielding genotype had the greatest below ground biomass at both sites.
• Below ground biomass allocation differed by up to 10% between genotypes and 94% between sites.
• Environment e.g. wind speed and soil characteristics affected biomass allocation.

## 1 Introduction

Willows (*Salix* spp.) grown as short rotation coppice (SRC), are being developed as sources of biomass for the production of bioenergy, biofuels and high value products for the chemical industries especially across Europe, north-eastern and mid-western USA and Canada [1–4]. Advantages of using SRC willow for biomass production include fast growth and high biomass production, ability to re-sprout after multiple harvests, ease of vegetative propagation from dormant woody cuttings, a wide genetic base for breeding and a positive greenhouse gas (GHG) balance [1,4–6].

Willow is a perennial shrub with an extensive below ground root system, which increases in size as the plant ages [7,8]. The root system stores essential carbohydrate reserves needed primarily for respiration and growth, and, to a lesser extent, symbiotic associations and exudation [9,10]. Mobilisation of carbohydrate reserves in the roots and cut stump are particularly important for re-sprouting of shoots after coppicing [9,11–14].

Estimates of below ground biomass production and the allocation of total plant biomass to above and below ground pools in willow are limited as roots are notoriously difficult and time consuming to sample. Studies are generally restricted to a small number of genotypes sampled at a few points during growth, often on roots and shoots of different ages which makes comparisons problematic. Rytter [15] studied roots of 1–3 year old plants of *Salix viminalis* (a popular species for biomass) in lysimeters. Annual net primary productivity of root biomass increased from 1 to 3 years, but the actual allocation below ground declined each year from 25–30% to 10–12% of total biomass in 1- and 3-year old plants, respectively. Both values increased markedly if fine root turnover was included in the estimate. Matthews [8] measured root biomass of two *S. viminalis* varieties, sampling five single plants from two sites, four Bowles hybrid from one site and a single Gigantea variety from the second site. The age of the individual *S. viminalis* stools varied from 4 to 22 years old and the shoots were of 1–3 years in age dependent on the date of the last coppice. From these limited data it was estimated that investment below ground increased from 10 to 25 odt ha^−1^ over 25 years, and in contrast to Rytter [15] the allocation of total plant biomass to below ground also increased marginally. Weih and Nordh [16] predicted the root biomass allocation for six three-year old willow varieties grown in field trials in central and southeast Sweden and revealed significant variety variation in below ground biomass allocation of 10–20% of the total plant biomass [16]. Pacaldo et al. [7] looked at root volumes in a single willow genotype of 5, 12, 14 and 19 years of age, planted in the field across three locations. Below ground biomass increased with plant age, up until 14 years. However, the allocation of total plant biomass to the roots showed no difference with age.

Knowledge of below ground biomass in perennial crops such as willow is important for understanding the GHG balance of bioenergy systems, as the roots have considerable potential to contribute towards the carbon (C) sequestration potential of the crop [17–21]. Previous life cycle assessments (LCA) of willow have relied upon limited below ground data to estimate C sequestration into root systems [8,22] and although a few more detailed data sets now exist [7,18] considerable investigation is still needed to recognise the potential for the capture of C into this important biomass pool, as well as the surrounding soil.

Within all plants there exists a functional equilibrium of biomass, where additional biomass is allocated to an organ to take up the resource that is most limiting growth [23–25]. An understanding of these principles is founded in plant ecology, but they have many applications in agricultural research as allocation sets limits on biomass production and utilisation [24]. Allocation patterns can be affected by numerous environmental factors to varying extents including: light, nutrients, water, elevated atmospheric CO_2,_ temperature, salinity and mechanical perturbation, and have been reviewed by numerous authors using a plethora of data from environmental manipulation studies [e.g. [24–27]].

As roots are notoriously hard to sample it would be useful to predict root biomass from other growth traits which are easier to measure. Previously, leaf area (and the closely related leaf area index, LAI) have been demonstrated to be closely correlated to above ground biomass in poplar [e.g. [28–30]]. Weih and Nordh [16] demonstrated the same relationship in willow, showing leaf area from pot experiments to be a good predictor of biomass production in the field. Supporting this, Andralojc et al. [31] showed that total leaf area and above ground biomass were closely correlated in a diverse set of 11 willow genotypes. Fewer studies have considered the belowground biomass. Bouman and Sylliboy [32] demonstrated a positive correlation between root biomass and leaf area in 12 willow varieties, and Weih and Bussel [33] showed a positive relationship with above ground biomass. However, an inverse relationship between leaf area and below ground biomass has also been reported [16,34].

To understand how allocation patterns vary, we studied above and below ground biomass in a set of four diverse high yielding willow genotypes established in two replicated trials in contrasting climates in the UK. Repeated above and below ground biomass harvests were conducted over two successive short rotation cycles to test the following hypotheses:

1. Diverse willow genotypes will differ in below ground biomass production and allocation.
2. The below ground biomass can be predicted from relationships with other growth traits such as leaf area.
3. Below ground biomass allocation will be altered by the growth conditions (climate and soil).

## 2 Materials and methods

### 2.1 Plant material and field trials

Four genotypes of willow were selected for the study; for their individual pedigrees see Table 1. All genotypes are the results of Swedish and UK breeding programmes, currently only Endurance is not yet a registered variety [35]. The genotypes were chosen because they produced good yields when tested in trials at multiple locations across UK and Ireland [35] (Table 1). However, they differ strongly in morphology e.g. leaf area index (LAI), stem number and canopy height, suggesting they achieve the high yields via diverse routes. Peak LAI was recorded in July 2011 using a SunScan Canopy Analysis system, type SS1 (Delta-T Devices Ltd., Cambridge, UK). Canopy height and stem number are shown for end of the growing season (October 2011). Canopy height was measured from the soil surface to the tip of the tallest stem using a telescopic measuring pole (Senshin Industry Co., LTD, Osaka, Japan 538–0041) (Table 1).

Two identical field trials were established in the UK at Rothamsted Research, Harpenden, southeast England (51.82 °N, 0.38 °W) and the Institute of Biological, Environmental and Rural Sciences (IBERS), Aberystwyth, west Wales (52.41 °N, 4.01 °W) during May 2009 as part of the BBSRC Sustainable Bioenergy Centre [36]. Each trial was a randomised block design, containing four blocks with one plot of each willow genotype per block. The plots measured 26.4 × 8.5 m and contained 374 plants in 11 double rows, equating to a planting density of 16, 667 plants ha^−1^ which is marginally higher than the industry recommendations of 15,000 plants ha^−1^ [37]. The plots had spacing's of 0.5 m between the plants in the rows and 0.8 m between the narrow rows and 1.6 m between the wide rows. The willows were planted as 20 cm cuttings in March 2009 using material harvested from mature willow stands in January 2009. Endurance and Tora were sourced from trials at Rothamsted Research, whilst Resolution and Terra Nova were supplied by Murray Carter Ltd (Ingerthorpe Hall Farm, Markington, Harrogate, North Yorkshire, HG3 3PD). Within each willow plot, a double row was designated to non-destructive measurements and yield determination whilst the remainder of the plot was assigned to destructive sampling and guard plants. The trial was designed such that each pair of plants designated for destructive sampling and the outer edge of every plot was surrounded by guard plants of the same genotype. This arrangement ensured that the excavations of trees from the destructive sampling area didn't influence the growth of nearby trees used for destructive measurements at a later date or the growth of trees used for yield determination and collection of non-destructive trait data.

The soil at the Harpenden site is a silty clay loam (sand 13%, silt 62% and clay 25% [38]) whilst at the Aberystwyth site, the soil is a sandy silt loam (sand 41%, silt 51%, and clay 8%; unpublished data). The average bulk densities of the top 30 cm of the soils are 1.48 ± 0.03 g/cm^3^ at Harpenden and 1.09 ± 0.04 g/cm^3^ at Aberystwyth. The volumetric water content of the soils at saturation are 38% and 43%, the water released through natural drainage is 6.9% and 7.4% and the plant available water is 8.3% and 13.6% for the Harpenden and Aberystwyth sites respectively. Previous to the establishment of the BSBEC trials the Harpenden site was planted with wheat, whilst the Aberystwyth site was under grassland. These different land uses are reflected in the soil organic C content which is 1.3  ±  0.04% at Harpenden and 3.9  ±  0.1% at Aberystwyth. Both sites were ploughed before the willow was planted. During winter 2010 the stools were coppiced to promote increased shoot production per stool. Ammonium nitrate was applied at a rate of 60 kg N ha^−1^ during spring 2010 and herbicide (Weedzol-TL) was applied at a rate of 20 l ha^−1^ to suppress weeds; weeds were further controlled by mowing between the rows throughout the season. Stand survival was excellent at both locations with only 0–4% losses per plot.

### 2.2 Climatic measurements

Meteorological conditions were monitored using specially installed weather stations on each trial (Campbell, Scientific Ltd, Loughborough, UK). Each was fitted with a CR1000 data logger, an AM25T multiplexer and sensors to record air temperature and humidity at 150 cm above the soil surface. Soil moisture content and soil temperature was monitored at depths of 10, 30 and 60 cm, but only data from the 10 cm sensors are presented here as the same trends between sites were evident at each depth. In addition, at Aberystwyth, sensors were installed to record incoming photosynthetically active radiation (PAR), rainfall, and wind speed. At Harpenden these variables were obtained from the electronic Rothamsted Archive (e-RA) which holds hourly data from the central Rothamsted meteorological site (Rothmet) located 1.7 km from the BSBEC trial across flat ground. At the dedicated weather stations sensors were triggered to take measurements every 15 min and hourly values were returned. For volumetric water content (*θ*), soil temperature and relative humidity monthly mean values were calculated as the average of daily means over each month. The maximum and minimum air temperatures were obtained from the absolute maximum and minimum temperatures for each day, averaged over each month. Wind run is the hourly average wind speed (m s^−1^) converted to km hr^−1^ and summed to give daily wind run (km day^−1^).

### 2.3 Total harvestable yield

The willow plots were harvested on a two year rotation after a 1st year cut back in 2010, followed by yield harvests during January 2012 and January 2014 when the crop was dormant. Within each plot all stems from the 26 plants in the designated yield double row were cut. The stems were weighed, then chipped and a subsample collected, weighed, and oven-dried at 80 °C to a constant weight. The dry weight of the chipped sample was used to calculate the yield per plot of the harvested double row which was then scaled up to tonnes of dry matter per hectare per year (t DM ha^−1^ yr^−1^).

### 2.4 Above and below ground biomass measurements

Throughout the two rotations the above and below ground biomass was determined in the destructive measurements area of the plot which is separate from the area used to measure the total harvestable yield (section 2.3). Fifteen measurements of the above and below ground biomass occurred over the two rotations with 11 harvests during the first rotation and four during the second (Table S1). The harvests were co-ordinated with particular plant phenological stages, especially during the 2011 season (Table S1). A pair of neighbouring plants (two plants) from a double row were randomly selected from the designated destructive sampling area in each plot. All stems were cut at 10 cm from the soil surface and (if present) leaves were separated. The stems were weighed, then chipped and a subsample collected, weighed, and oven-dried at 80 °C to a constant weight. At the destructive samplings when plants were in leaf (10 occasions), the total leaf area per plant was calculated by scanning a representative subsample using a WinDias Image analysis system (WD3, Delta-T-Devices, Cambridge, UK). The subsample and remaining leaves were oven-dried at 80 °C to a constant weight and total leaf area (*LA*_*total*_) was calculated as:

$$LA_{total}=\left(\frac{LA_{sub}}{LDW_{sub}}\right)LDW_{total}$$

where *LA*_*sub*_ is the subsample leaf area (cm^2^), *LDW*_*sub*_ the leaf subsample dry weight (g) and *LDW*_total_ the dry weight (g) of all the leaves.

To sample the below ground biomass a quadrat of 50 × 120 cm was placed around each of the cut plants so that half of the gap to neighbouring plants fell within it. The quadrat area was derived from the area dedicated to each individual plant in the plot. It was considered that neighbouring plants may root in that area just as the individual planted there may root outside of the area. The whole area was excavated to a depth of 30 cm to recover the greater part of the below ground biomass. Previous work has shown that 80–85% [39] and up to 92% [7] of below ground biomass is located within in the upper 30 cm of the soil profile. After excavation the below ground biomass was separated into the below ground stool, above ground stool, coarse roots and fine roots. The below ground stool refers to the initial stem cutting planted in the soil which increases in size and develops roots as the plant ages. The above ground stool is the stump that remains above the soil after harvesting and contains the buds for the next season growth [7]. The above ground stool was separated from the below ground stool by cutting the stool at soil level. The coarse roots were cut from the below ground stool at the point of emergence; roots with a diameter ≥2 mm were classified as coarse roots and those <2 mm as fine roots [39]. The soil from the trench was raked through to collect any remaining visible root material that had become detached from the main stool. No sieving and washing of soil from the trench took place as the number of plants (32) and harvests (15) made it impractical, therefore we will have only retained a proportion of the fine root material. The stool and root material were washed clean of soil and oven-dried at 80 °C to a constant weight. The total above ground biomass (leaves, stems and above ground stool) and total below ground biomass (below ground stool and roots) were then then scaled up to tonnes of dry matter per hectare (t DM ha^−1^).

### 2.5 Fine root biomass

#### 2.5.1 Collection of soil cores

As fine roots are an important component of the below ground biomass [7,15,40] and fine root volumes were not accurately assessed using the methods described in section 2.4, an in-depth sampling of fine root biomass was carried out to coincide with the destructive harvest during the second rotation in July 2013 at both field sites. This was when there was suitable soil drying to allow access for ease of extraction of soil cores. Fine roots were sampled from Endurance and Resolution plots only as these genotypes provided the best representations of the contrasting growth habits. Before any excavations of below ground biomass occurred (section 2.4), three soil cores were taken from each plot, focussed around one plant of the pair to be destructively sampled. A corer containing an inner sleeve that could be split longitudinally (diameter 70 mm; length 1 m) was driven into the soil using a hydraulic jackhammer and extracted using a tripod ratchet. The first core was collected between the plant pair, i.e. in the centre of the narrow row; the second core was collected one quarter of the way across the wide row, i.e. 40 cm from the selected plant; and the third core was collected half way across the wide row i.e. 80 cm from the selected plant. The cores were kept whole, wrapped in plastic and stored in a freezer at −20 °C until processing to prevent decomposition of fine roots.

#### 2.5.2 Processing of soil cores

The frozen cores were thawed for processing and divided into five depth intervals: 0–10, 10–20, 20–30, 30–50 and 50–100 cm. Each interval was then split in half vertically and the fresh weight of each half recorded. One half was reserved to assess soil properties (not reported here) whilst the second was used for root measurements. Roots were separated from the soil by rinsing over a 1 mm sieve. After washing, roots were refrigerated at 4 °C in jars containing 20% ethanol for up to 2 weeks until they were floated on water in a shallow tray and scanned on a flatbed scanner (EPSON^®^ Expression^®^ 1600, Long Beach, CA, USA). The images obtained from the scanner were analysed using the *WinRhizo*^*TM*^ root scanning software program (2002a Pro, Regent Instruments Inc., Canada) to determine the total length, volume, surface area and diameter of roots in the five depth intervals. The total root length was corrected for the volume of the interval from which the roots were extracted to give the root length density (RLD, m cm^3^).

### 2.6 Statistical analysis

Statistical analyses were carried out using the computing package R [(version 3.0.1) Copyright^©^ 2013, The R Foundation for Statistical Computing] with *P* = 0.05 as the critical level of significance. Data from rotation one and two were treated separately. Above and below ground biomass data were analysed using a linear mixed effects model (lme) to test for differences between genotype, site and sampling date and any interactions therein. Genotype, site and sampling date were designated as fixed effects and block and sampling date as random effects. For the annual yield there was one harvest per rotation, therefore the (lme) analysis considered genotype, site and genotype × site only, and included block as a random effect. RLD was measured in two genotypes; the (lme) was used to uncover differences between genotype, site and depth interval and test for interactions between these variables (genotype × site × depth), block and core number were both considered as random effects. For all analyses any data that did not meet the conditions of normality were transformed.

## 3 Results

### 3.1 Climate

**Fig. 1:**
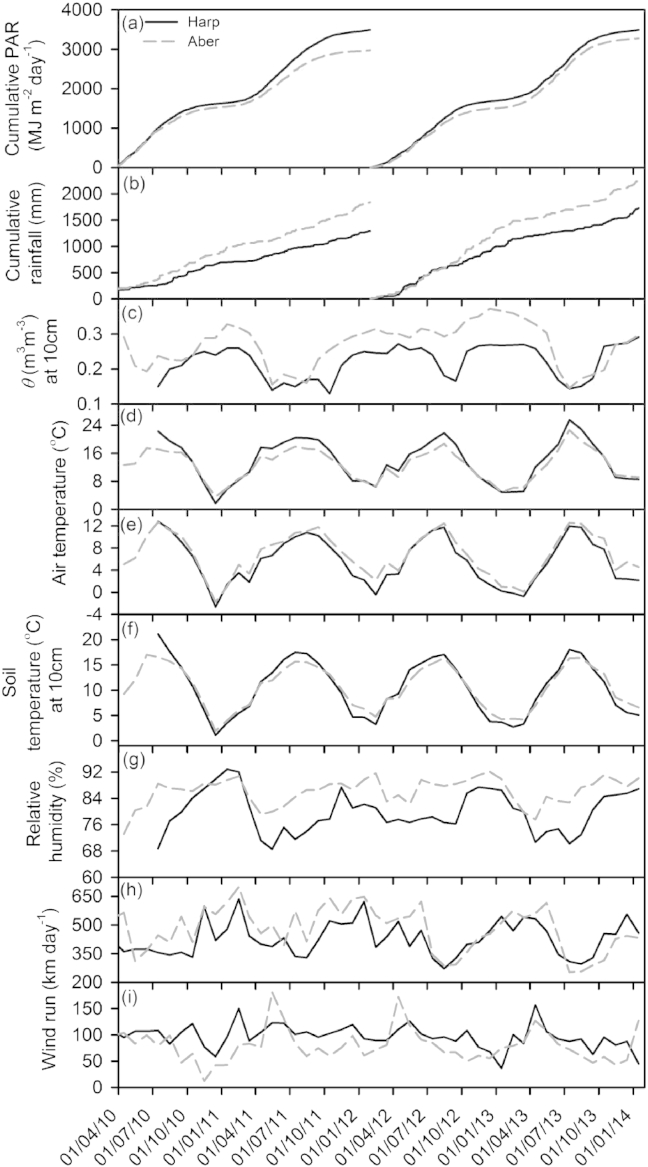
Climatic data from 2010 to 2014 at Harpenden (Harp) and Aberystwyth (Aber). Cumulative photosynthetically active radiation (PAR) (a), Cumulative rainfall (b), volumetric water content (*θ*) at 10 cm depth (c), average monthly maximum air temperature (d), average monthly minimum air temperature (e), average soil temperature at 10 cm depth (f), average relative humidity (g), monthly maximum wind run (h), and monthly minimum wind run (i). Cumulative PAR and rainfall are calculated for each rotation (rotation one = 2010–2012, rotation two = 2012–2014).

Climatic data are summarised over the two crop rotations (2010–2014) for the two sites Harpenden and Aberystwyth (Fig. 1). Cumulative photosynthetically active radiation (PAR) was greater at Harpenden compared to Aberystwyth. However, the accumulation was greater during the first than the second rotation, with Harpenden receiving 17% and 7% more PAR than Aberystwyth respectively (Fig. 1a).

Aberystwyth is a wetter site than Harpenden, as shown by the greater cumulative rainfall during rotation 1 (42%) and rotation 2 (31%) (Fig. 1b). The level and distribution of rainfall is reflected in the volumetric soil water content (*θ*). Overall there was more water available at Aberystwyth but a water deficit occurred at both sites during summer 2010, 2011 and 2013 (Fig. 1c) which reduced growth rates of the willow [41].

The average air temperature showed little difference between the two sites (data not shown). However, Harpenden experienced a greater range of temperatures, with higher maxima and lower minima (Fig. 1d and e). For example, during July 2011 and 2012 the average maximum temperatures at Harpenden were 3.1 and 2.4 °C higher, respectively. Whilst during winter, temperatures reached an average minimum of −2.7 °C in 2011 and -0.4 °C in 2012 at Harpenden, yet did not drop below −1.7 (2011) and 2.2 °C (2012) at Aberystwyth. The patterns in air temperature were reflected in the soil temperatures; during the summer, average monthly soil temperatures were higher at Harpenden, whilst during the winter average monthly soil temperatures were lower (Fig. 1f).

Reflecting the warmer temperatures and lower rainfall, average monthly relative humidity (RH) was significantly lower at Harpenden compared to Aberystwyth, with the largest discrepancy between sites occurring in the summer months. At Harpenden humidity reached a minimum of 68%, 77% and 70% during 2011, 2012 and 2013 respectively, whilst at Aberystwyth humidity did not drop below 79% over the three years (Fig. 1g).

Average wind run shows little difference between sites (data not shown). However, the range of maximum and minimum monthly wind run was greater at Aberystwyth, especially during the first rotation (Fig. 1h and i). During 2011 there were only 3 months at Aberystwyth where maximum wind run did not reach 500 km day^−1^, compared to nine out of 12 months at Harpenden. In the same year wind run dropped to a minimum of 100 km day^−1^ at Aberystwyth for ten of the twelve months whilst wind run was only less than 100 km day^−1^ for 2 months at Harpenden (Fig. 1h and i).

### 3.2 Total harvestable yield

**Fig. 2:**
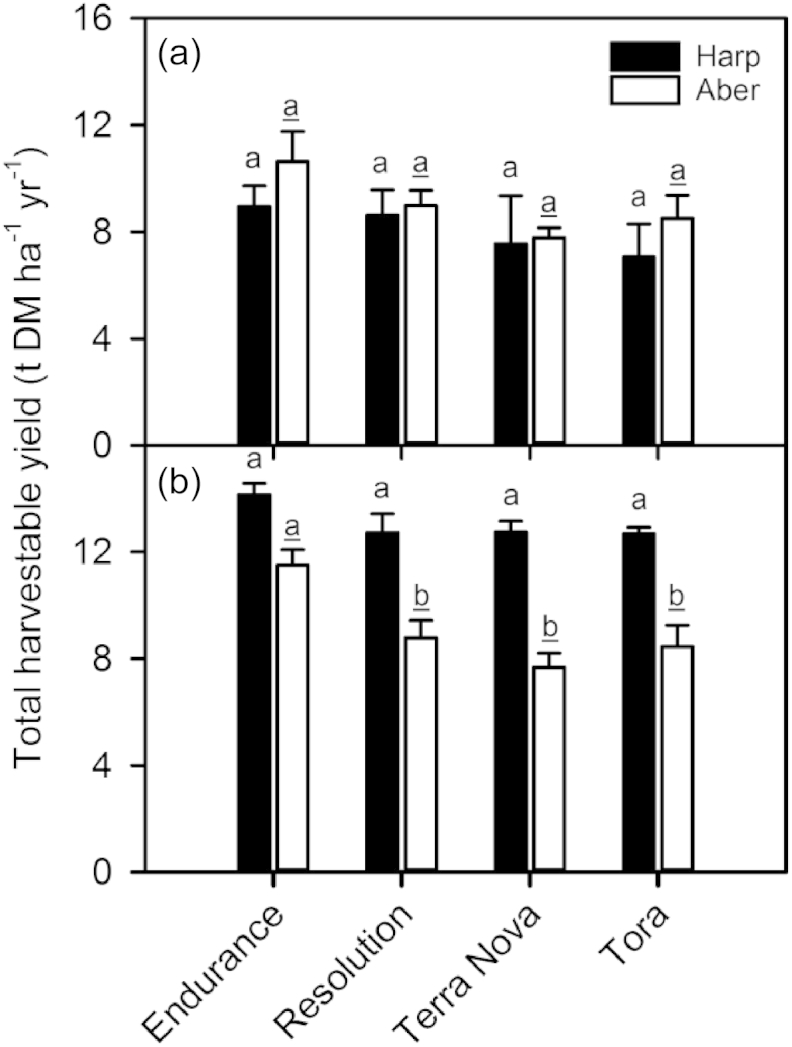
Total harvestable yield (tonnes dry matter per hectare per year (t DM ha^−1^ yr^−1^)) for the four willow varieties grown at Harpenden (Harp) and Aberystwyth (Aber) during the first rotation (a) and second rotation (b). Data are means + SE of four replicates. Different letters indicate significant differences between the genotypes (*P* < 0.05), with differences at Harpenden indicated by the lower case letters and differences at Aberystwyth the underlined lower case letters.

Total harvestable yield was measured at the end of the first rotation in 2012 and the end of the second rotation in 2014 (Fig. 2). During the first rotation there was no significant difference in yields between the four willow genotypes and between the two sites (Fig. 2a; Table 2). However, during the second rotation yields were significantly greater at Harpenden compared to Aberystwyth and there were significant differences between genotypes (Fig. 2b; Table 2). A lack of interaction between genotype and site shows that the ranking of the genotypes in terms of yield was similar at the two locations (Fig. 2b; Table 2). Endurance had the greatest yield reaching 14.1 and 11.5 t DM ha^−1^ yr^−1^ at Harpenden and Aberystwyth respectively, whilst the remaining 3 genotypes displayed similar yields of 12.7 t DM ha^−1^ yr^−1^ at Harpenden and 7.7–8.8 t DM ha^−1^ yr^-1^at Aberystwyth.

### 3.3 Biomass allocation

**Fig. 3:**
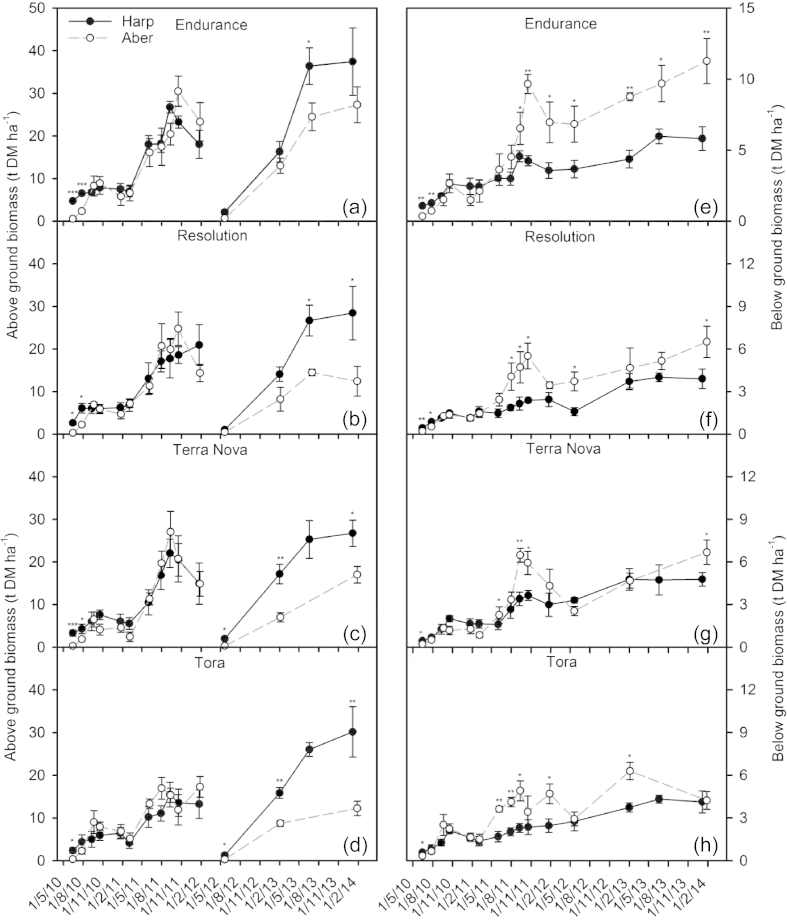
Dynamics of the above ground biomass (a–d) and below ground biomass (e–h) over two rotations for the four willow varieties: Endurance, Resolution, Terra Nova and Tora, grown at Harpenden (Harp) and Aberystwyth (Aber). Data are means ± SE of four replicates. Significant differences between the sites are indicated by ***=<0.001, **=<0.01 and *=<0.05. Rotation 1 = 2010–2012, rotation 2 = 2012–2014.

Total above ground biomass increased during the first rotation, from the first destructive sampling in 2010 to the final sampling at the start of 2012, for all genotypes at both sites (Fig. 3a–d). Biomass peaked at the end of August in each year (2010 and 2011) and then showed an overwinter decline, due to the loss of leaves and small branches (Fig. 3a–d). Genotypes showed significant differences, with Endurance having the greatest above ground biomass at all sampling times and Tora the smallest, whilst Resolution and Terra Nova were intermediate (Fig. 3a–d; Table 2). No significant differences in above ground biomass were found between sites (Table 2).

During the second rotation, above ground biomass increased from the first harvest in May 2012 to the final harvest at the start of 2014. The same pattern of significant difference between genotypes was found as described previously for the first rotation (Fig. 3a–d; Table 2). A strong difference between sites was detected in the second rotation, with genotypes displaying a significantly greater above ground biomass at Harpenden compared to Aberystwyth (Fig. 3a–d; Table 2).

Below ground biomass was lower at Aberystwyth for the first destructive sampling in June 2010 (Fig. 3e–h). However, throughout 2010 and early 2011 it increased more rapidly at the Aberystwyth site, until it was significantly greater in all genotypes for the majority of the remaining sampling dates (Fig. 3e–h; Table 2). In fact, by the final sampling date, during January 2014, below ground biomass was up to 94% greater in Endurance growing at Aberystwyth compared to Harpenden. Below ground biomass followed a seasonal cycle, with a reduction over the winter months and this was more apparent at Aberystwyth due to the greater mass. Significant differences were detected between genotypes; below ground biomass was greatest in Endurance (reaching a maximum of 675 g at Aberystwyth) and was similar for the other 3 genotypes which weighed a maximum of ∼400 g each at Aberystwyth (Fig. 3e–h; Table 2).

**Fig. 4:**
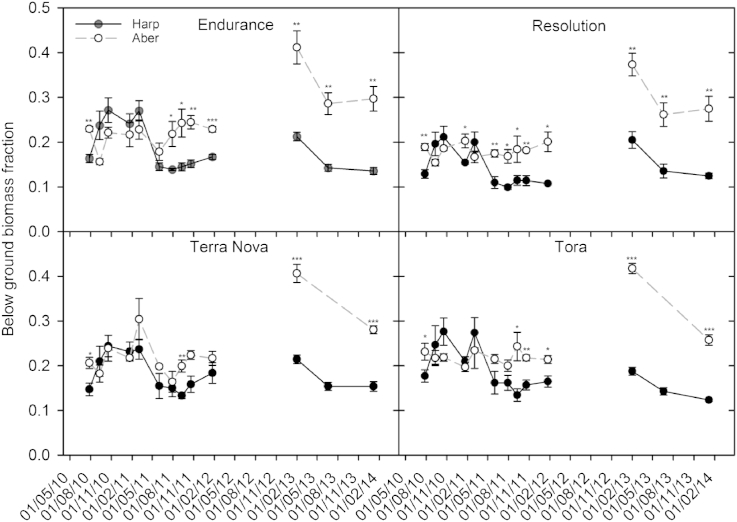
Fractional allocation of biomass to the below ground over two rotations for the four willow varieties: Endurance, Resolution, Terra Nova and Tora, grown at Harpenden (Harp) and Aberystwyth (Aber). Data are means ± SE of four replicates. Significant differences between the sites are indicated by ***=<0.001, **=<0.01 and *=<0.05. Rotation 1 = 2010–2012, rotation 2 = 2012–2014.

The allocation of biomass to the below ground is shown in more detail in Fig. 4. The below ground biomass fraction is similar at the two sites until July 2011 when they start to diverge and the fraction of biomass allocated below ground begins to increase at the Aberystwyth site. This significant difference between sites remains until the end of the first rotation and throughout the second rotation (Fig. 4, Table 2). Significant differences between genotypes were found throughout both rotations (Table 2). However, within site genotypic differences were not large with the proportion of biomass allocated below ground differing by a maximum of 10%. Generally, Endurance allocated the greatest proportion of biomass below ground and Resolution the least.

### 3.4 Fine root biomass

**Fig. 5:**
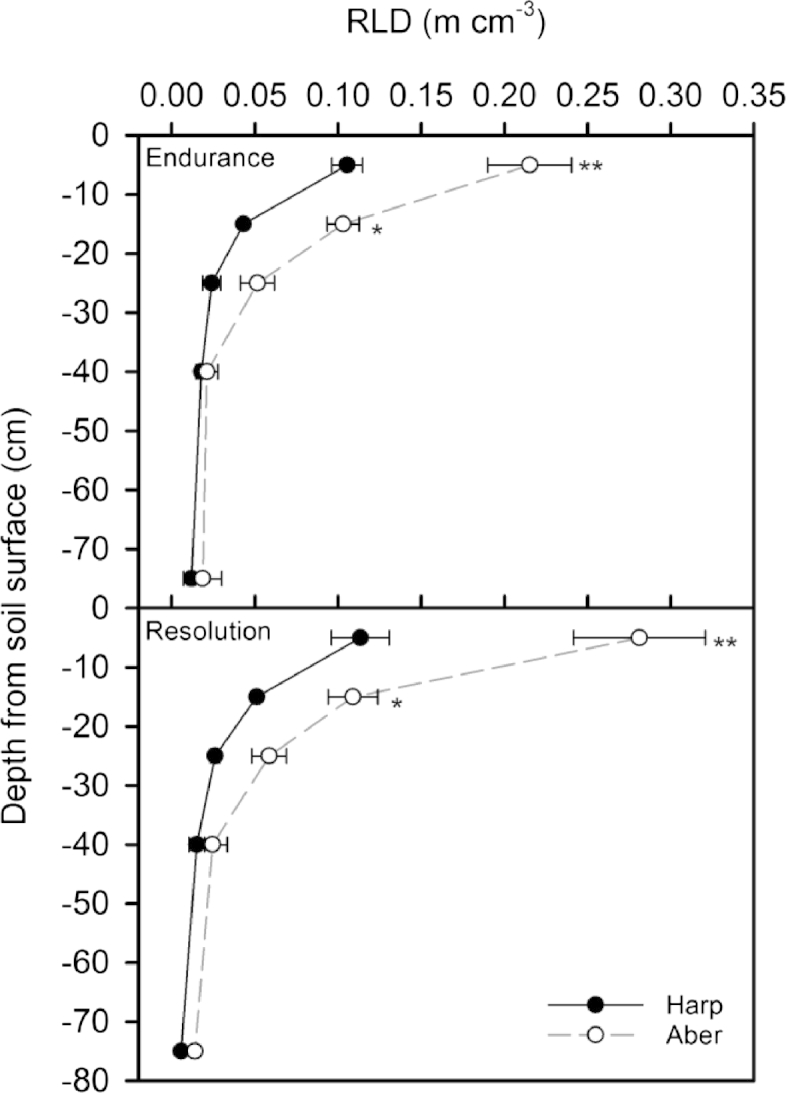
Root length density (RLD) at five depths for the two willow varieties: Endurance and Resolution grown at Harpenden (Harp) and Aberystwyth (Aber). Data are means ± SE of four replicates. Significant differences between the sites are indicated by **=<0.01 and *=<0.05.

**Fig. 6:**
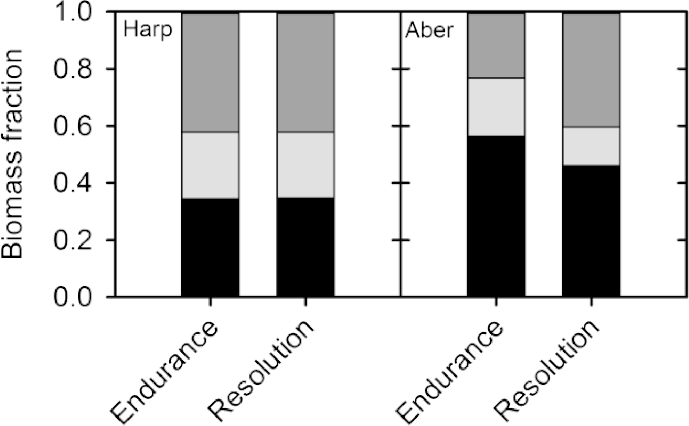
The fraction of below ground biomass allocated into the belowground stool (black), coarse roots (pale grey) and fine roots (dark grey) for the two willow varieties: Endurance and Resolution grown at Harpenden (Harp) and Aberystwyth (Aber).

Fine root volumes were measured as the root length density (RLD). RLD declined with increasing depth from the soil surface for both genotypes (Endurance and Resolution) at the two sites; in fact the majority of fine roots were concentrated in the first 0–10 cm of soil (Fig. 5). Genotypes did not show a significant difference in RLD, but there was a significant difference between sites with both genotypes at Aberystwyth having a much greater RLD (Table 3; Fig. 5). For example, at Aberystwyth, in the first 0–10 cm of soil, there was a 103% and 147% greater volume of fine roots for Endurance and Resolution respectively. This difference remained until depths of 40 cm and deeper when there was no difference in root volumes between sites (Fig. 5). Using the fine root data, alongside the below ground biomass data collected at the June 2013 destructive sampling the fraction of biomass allocated into the different below ground components was estimated (Fig. 6). Interestingly, there is a trend towards greater allocation into the belowground stool and coarse roots compared to the fine roots for the genotypes growing at IBERS, particularly for Endurance.

The fine root data was used to estimate how much biomass was lost from the below-ground with our sampling method. We found losses of 50–73 g of fine roots when comparing those recovered by raking through the pits to those calculated from soil cores. This equates to an underestimation of the below ground biomass (fine roots) of 16–24%.

### 3.5 Leaf area and below ground biomass

**Fig. 7:**
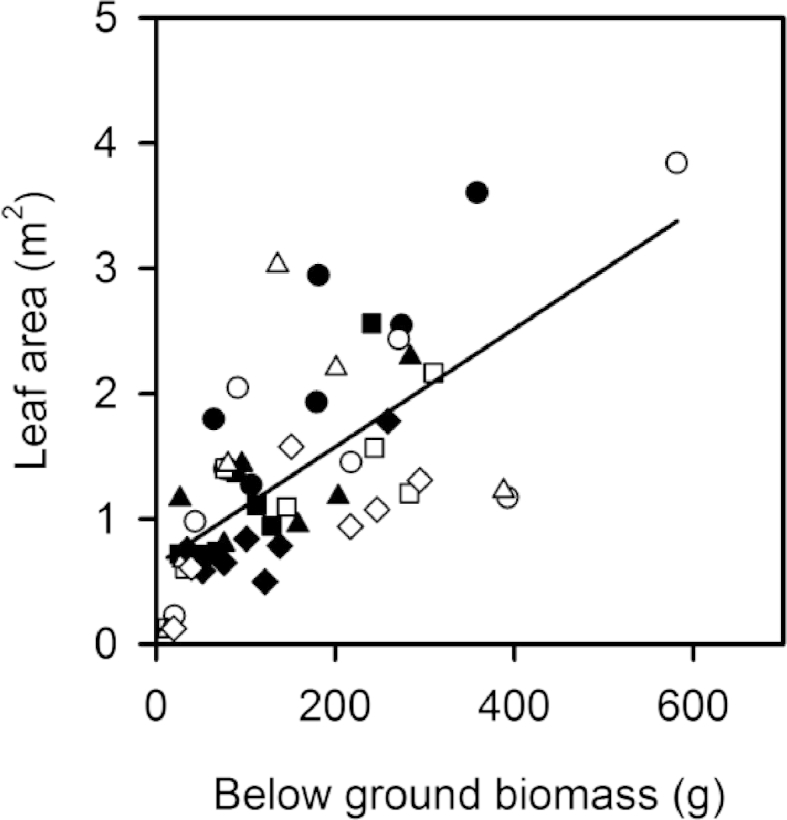
Regression slope for the relationship between leaf area and below ground biomass (y = 0.0047x + 0.6317; F = 43.9, *P* < 0.001, R^2^ 0.49). Data are from the seven destructive harvests which occurred during the main growing season (June, July and Sept 2011 and 2012, and June 2013). Harpenden is the closed symbols and Aberystwyth the open symbols. Endurance (circles), Resolution (squares), Terra Nova (triangles) and Tora (diamonds).

As expected, total leaf area was significantly different between genotypes across both rotations (Table 2; Fig. S1). For example, during June 2011, leaf area was the largest in Endurance reaching 2.9 m^2^ at Harpenden and 2.4 m^2^ at Aberystwyth, followed by Terra Nova whose leaf area was 1.4 m^2^ and 2.2 m^2^ at Harpenden and Aberystwyth respectively. Resolution and Tora showed the smallest leaf areas of 1.3 m^2^ and 1.5 m^2^ for Resolution and 0.8 m^2^ and 1.1 m^2^ for Tora at Harpenden and Aberystwyth respectively (Fig. S1). A significant difference in leaf area between the two sites was found in the first rotation but not the second (Table 2). Leaf area showed a positive correlation with the total below ground biomass (Fig. 7).

## 4 Discussion

### 4.1 Allocation patterns differ between genotypes

We found significant differences in biomass allocation between the four willow genotypes included in this study, demonstrating that capacity to manipulate the trait exists. The variation between genotypes in below ground biomass allocation was up to 10% and this is within the range of variation found by Weih and Nordh between 6 different willow clones [16]. Furthermore, we found that Endurance, the genotype which had the largest below ground biomass, had equal or greater above ground yields than the other three genotypes. This shows that in Endurance, yield was not compromised by increased biomass partitioning to the below ground organs. This study used a limited number of willow genotypes and there are circa 400 different species (depending on the classification used), thus there is a large resource in which different allocation patterns could be identified [1].

Bouman and Sylliboy [32] looked at biomass allocation in 12 different willow varieties, finding large differences in root volumes which were positively correlated with leaf area. Similarly, in this study, we found that Endurance had the largest leaf area as well as the greatest below ground mass, and that overall leaf area was positively correlated with root biomass. However, unlike the genotypes studied here, Weih and Nord [16,34] demonstrated an inverse relationship between leaf area and root biomass in six willow clones, suggesting that increased shoot productivity with increasing leaf area occurred at a cost of reduced below ground biomass allocation. As an evolutionary adaptation it makes sense that a genotype with a high resource acquiring surface area should have a correspondingly large surface area below ground to acquire water and nutrients [42]. Leaf area ratio (leaf area per unit plant mass) and root length ratio (root length per unit plant mass) have both been positively (and strongly) correlated with relative growth rate in nine Boreal tree species [42]. Above ground growth rate between the March 2011 and June 2011 harvests was greatest in Endurance at both sites reaching 7.1g day^−1^ and 6.1g day^−1^ at Harpenden and Aberystwyth respectively (Fig. S2). Therefore, Endurance surpassed a weight of 1 kg plant^−1^ by June 2011 during the first rotation at both sites, whereas all other genotypes took until at least August. Therefore, it appears that positive relationships between leaf area, root biomass and growth rate are also present in willow. These relationships also present the possibility that canopy area, which can be measured non-destructively, could be used as an easier predictor of below ground biomass.

### 4.2 Environmental influence on partitioning

Our data from the Harpenden site agree with the study by Rytter [15], which showed that the below ground biomass of willow increased with plant age, but overall allocation below ground fell. In Endurance and Resolution at both sites, the fine roots were largely concentrated in the first depth interval of 10 cm, which also agrees well with other studies on the distribution of willow root biomass [7,20,39]. However, at the Aberystwyth site, below ground biomass was much greater in all genotypes, and allocation below ground tended to increase, especially during the second rotation. These between-site differences were much larger than the within site genotypic allocation differences. For Endurance, growing at Aberystwyth, the biomass of the coarse roots and below ground stool combined was up to 94% greater than that at Harpenden by the end of the second rotation. Furthermore, plants had double the volume of fine roots when growing at Aberystwyth compared to Harpenden. Differences in climate and soils need to be considered to help explain this unexpected discrepancy.

Low temperatures (below 18 °C [24]) can increase biomass allocation to roots as a range of plant functions are impaired [e.g. [43–45]]. Specifically, nutrient uptake and water uptake are slowed, and nutrient cycling rates in the soil are reduced [24,43,46]. Average temperatures were lower at the Aberystwyth site during the summer months but during spring and autumn when some root growth still occurred, temperatures were largely equal. Moreover, previous reports indicate only modest changes in allocation above 18 °C, suggesting only minimal adjustments might occur over the temperature range seen here [24].

It is unlikely that light levels can account for the observed differences in partitioning. Low light levels have been shown to increase allocation to above ground organs, specifically to leaves to increase production when carbon is limiting and to branches and stems to fuel height growth through gaps in the canopy [26,42,47]. However, the majority of these studies looked at very low light levels experienced by shade grown plants. In fact, allocation becomes saturated above 20 mol m^−2^ day^−1^ and the plants in this study were receiving well above this light level at both sites [24].

Rainfall was considerably lower at Harpenden than Aberystwyth for rotations 1 and 2. Reduced water availability can increase investment in roots, although this generally occurs when plants are subjected to severe drought stress and total biomass is severely reduced (by 4–6 times) [48–50]. Declines in total biomass of this extent were not seen in this study. Possibly, optimal partitioning does not operate when environmental challenges are relatively short-lived (e.g. unpredictable rain or drought events), as responding too quickly to shorter-lived stress may result in sub-optimal growth once water supply is returned and thus be disadvantageous in the long run [24,25].

Coarse root and the below ground stool were larger at Aberystwyth. Furthermore, the data suggests (for one genotype) that there is an increased allocation of biomass into these fractions relative to fine roots. Interestingly, although no reduction in stem height occurred stems were substantially thicker (Fig. S3). Both are common physiological responses to mechanical stimuli [51]. Wind speeds were considerably higher at Aberystwyth where the site is very exposed, and considerably more turbulent, reaching higher maximum speeds and lower minimum speeds. Larger root biomass increases the magnitude of the mechanical forces required to dislodge a plant from its substrate [51]. Coutand et al. [52] found that trees encased in an artificial shelter had poor root growth compared to trees exposed to natural/mechanical wind stimuli, and Whitehead [53] found that high root biomass was maintained at the expense of shoot biomass in sunflower subjected to increasing wind speeds. Furthermore, Sitka spruce subjected to artificial flexing showed significant increases in coarse root mass [54].

Investigations of the impacts of relative humidity (RH) on allocation are of renewed interest with respect to climate change, which is expected to bring increased rainfall and humidity to northern latitudes. With increasing humidity allocation below ground is predicted to increase, as demonstrated in silver birch and aspen in a humidity manipulation experiment [55]. In particular, fine root biomass and specific area of fine roots were enhanced under high humidity [55]. It is suggested that high RH could impede nutrient assimilation due to decreased evaporation and thus a decline in the transpiration stream that moves to the upper parts of a tree, and therefore increased investment in roots is required to meet growth demands [55,56]. RH was consistently higher at Aberystwyth, rarely falling below 80% even with greater wind speeds.

Nutrient availability is well documented as strongly influencing allocation [24], with a large increase in roots occurring at the expense of shoot biomass when nutrients are limiting. In willow Rytter [57] found that root volumes were greater under limited nitrogen (N), specifically the annual production of fine roots (<1 mm) in the N-limited treatment exceeded that of the unlimited treatment by 31%. Weih and Nordh [16] showed that the root biomass fraction of 6 willow varieties declined with the addition of fertilizer. Measures of soil nutrients at the Aberystwyth trial site are not available for this study but soils at Aberystwyth have been shown to contain a greater percentage of sand (41% compared to 13% at Harpenden) and are known to be very shallow in some areas where bedrock is reached at less than 50 cm depth. Pacaldo et al. [7] found a high root biomass and lower stem biomass in willows growing on a site with soil depths of 40 cm and occasional floods which wash out nutrients. It is feasible that a similar situation could be occurring in the willows growing at Aberystwyth, where rainfall is also higher.

Furthermore, soils at Aberystwyth have a lower bulk density and, due to the contrasting previous land uses (grassland vs. arable), have a higher organic carbon content of 3.9% compared to 1.3% at Rothamsted. Combined with a greater amount of plant available water this suggests that the soil is both more porous and better-structured at Aberystwyth, and these properties may encourage the proliferation of roots as the soil is likely to be easier to penetrate. Soils with a high clay content and high bulk density have been shown to have a greater potential to impede root growth [58] and this may have been a factor in the finer textured soils at Rothamsted.

Given the above considerations we conclude that differences in allocation patterns between the two sites arose from a combination of environmental influences primarily differences in wind speed, with humidity, and soil properties and potentially available nutrients having additional impact.

### 4.3 Impact of allocation patterns on C accumulation potential

Carbon-sequestration potential cannot be evaluated from this study as long-term fine root dynamics were not measured [15,18], nor were mycorrhizal associations or soil microbial processes studied [59–61]. However, we can give an indication of the C accumulation potentials of the different willow genotypes using our measurements of above and below ground biomass from the end of the first and second rotation (January 2012 and 2014). C content was not measured directly, but if we assumed similar levels to previous studies (e.g. Rytter [18] used a common C-concentration of 500 mg g^−1^ biomass [62]) we can derive C accumulation levels in the crop of 0.79–1.17 t ha^−1^ year^−1^ below ground and 5.21–6.93 t ha^−1^ year^−1^ above ground, with the greatest potentials for both pools being in Endurance.

We also found that allocation varies strongly with environment and differences between sites were greater than the within-site genotypic differences. Therefore, not only does yield vary with growing environment but so does the below ground C accumulation potential. For example, during January 2014, the harvestable yields of Endurance were 20% less at Aberystwyth compared to Harpenden, but simultaneously the below ground biomass was 94% greater at Aberystwyth, demonstrating a greater capacity for C accumulation at this site.

Plants with desirable allocation patterns could be used to enhance C accumulation and this could be further enhanced by manipulating biomass allocation patterns through genetic screening, selective breeding and management [17,63–66]. Alternatively, some of the below ground growth could be reduced to increase above ground yield to meet growing energy demand. However, in this latter case care has to be taken not to impact regrowth potential [11]. To exploit such traits we need a better understanding of source: sink relationships and a more systems-based approach since it is unlikely that partitioning is under the control of a single gene, but rather an orchestrated response of multiple genes to environmental pressure and stimuli [24].

### 4.4 Rotation length and planting density

This study used a two-year rotation when traditionally a three-year rotation has been employed for willow SRC [37]. However, the willows used in this study are commercially grown and sufficiently high yielding to be managed within a two-year rotation, which indeed is what some UK commercial growers are now practicing for high yielding varieties. Earlier studies looking at the effects of changed harvesting frequency are mixed with some showing increased yields and others diminished [67,68], although these studies were conducted on older varieties so results may not be applicable here. To the authors knowledge no publications exist on the effect of harvesting frequency on biomass allocation. Our view is that, potentially trees on a 3–4 year cycle would have a greater below ground biomass as there would be less demand for the belowground reserves to be used for regrowth after coppice.

The planting density of 16,667 plants ha^−1^ used in this study is marginally higher than the industry standard of 15,000 plants ha^−1^ [37]. Bullard et al. [69] presented limited data on biomass partitioning which suggests greater below ground allocation at densities of 111,000 plants ha^−1^ compared to 10,000 plants ha^−1^. Similarly, both [70] and [71] showed increasing below ground biomass allocation at high densities and related it to smaller tree size or resource limitation [71]. However these studies all look at much larger increases in density than our deviation from the current recommended planting density. Comparing the effects of rotation length and density are both aspects worthy of future study.

## 5 Conclusions

Our study has demonstrated that different biomass allocation patterns exist in a limited number of (commercially grown) willow genotypes and that high below ground biomass does not preclude high above ground yields. Furthermore, we found that below ground biomass could be predicted from the leaf area which paves the way for the development of quicker methods to assess below ground biomass. Stronger than genotypic differences, changes in climate and resource availability were found to have a significant impact on biomass allocation patterns. At Aberystwyth it is hypothesised that high wind speeds caused mechanical stimulation which increased investment below ground to anchor the crop. Alongside this, the soil properties at Aberystwyth, combined with high humidity which can act to slow N translocation in the crop, may have favoured further investment in roots. Information from this study could be used to inform breeding to favour increased C accumulation, increased biomass yield, or potentially both.

## Figures and Tables

**Table 1:** Pedigrees and basic phenotypic characteristics of the four willow varieties.

Parents						
Variety	♀	♂	Yield range^a^ (t DM ha^−1^ yr^−1^)	Peak LAI^b^	No. stems	Canopy height (cm)
Endurance	*S. redheriana*	*S. dasyclados* ‘77056’	9.6–14.6	6.3 ± 0.3	13 ± 0.6	353 ± 21
Resolution	(((*S.viminalis* ‘N81102’ × *S.vimianlis* ‘L830201’) ‘Jonrunn’) × ((*S. schwerinii ‘*L79069’ × *S. viminalis* ‘Orm’)’Bjorn) ‘SW930812’)	((*S.viminalis* ‘Pavainen’) × ((*S. schwerinii ‘*L79069’ x *S. viminalis* ‘Orm’)’Bjorn) ‘Quest’)	8.1–14.1	4 ± 0.1	7 ± 0.2	476 ± 22
Terra Nova	((*S. viminalis* 'Bowles Hybrid' × *S. triandra* 'Brunette Noire') ‘LA940140’)	*S. miyabeana* 'Shrubby'	6.5–10.4	4.8 ± 0.1	13 ± 0.7	394 ± 35
Tora	*S. schwerinii* 'L79069′	((*S. viminalis* × *S. viminalis*) ‘Orm’)	9.1–13.1	4.1 ± 0.1	9 ± 0.5	424 ± 5

a Values are taken from Ref. [29] which reports yield data from trials at multiple locations across UK and Ireland across two SRC rotations.

b LAI = Leaf area index.

**Table 2:** Linear mixed effects model for annual yield, above ground biomass, below ground biomass, below ground allocation and leaf area for the four willow varieties over two rotations, testing for the effects of genotype, site, sampling date and any interactions therein. Significance levels were set at *P* < 0.05 and n.s is not-significant. Annual yield data is from a single time point so sampling date was not included in the analysis (n/a).

Rotation 1	Annual yield		Above ground biomass		Below ground biomass		Below ground allocation		Leaf area	
	*n*	*P*	*n*	*P*	*n*	*P*	*n*	*P*	*n*	*P*
Genotype	4	n.s	4	<0.001	4	<0.001	4	<0.001	4	<0.001
Site	2	n.s	2	n.s	2	<0.05	2	<0.05	2	<0.01
Date	–	n/a	11	<0.001	11	<0.001	11	<0.001	8	<0.001
Genotype × site	8	n.s	8	<0.05	8	<0.05	8	n.s	8	<0.01
Genotype × date	–	n/a	44	n.s	44	n.s	44	n.s	32	n.s
Site × date	–	n/a	22	<0.001	22	<0.001	22	<0.001	16	<0.001
Genotype × site × date	–	n/a	88	n.s	88	n.s	88	n.s	64	n.s

Rotation 2	Annual yield		Above ground biomass		Below ground biomass		Below ground allocation		Leaf area	
	*n*	*P*	*n*	*P*	*n*	*P*	*n*	*P*	*n*	*P*
Genotype	4	<0.001	4	<0.001	4	<0.001	4	<0.01	4	<0.001
Site	2	<0.001	2	<0.001	2	<0.01	2	<0.001	2	n.s
Date	–	n/a	4	<0.001	4	<0.001	4	<0.001	2	<0.001
Genotype × site	8	n.s	8	n.s	8	<0.01	8	n.s	8	n.s
Genotype × date	–	n/a	16	n.s	16	n.s	16	n.s	8	n.s
Site × date	–	n/a	8	<0.01	8	n.s	8	n.s	4	n.s
Genotype × site × date	–	n/a	32	n.s	32	n.s	32	n.s	16	n.s

**Table 3:** Linear mixed effects model for root length density (RLD) for the two willow varieties, Endurance and Resolution, testing for the effects of genotype, site and depth and any interactions therein. Significance levels were set at *P* < 0.05 and n.s is not-significant.

	RLD	
	*n*	*P*
Genotype	2	n.s
Site	2	<0.001
Depth	5	<0.001
Genotype × site	4	n.s
Genotype × depth	10	n.s
Site × depth	10	n.s
Genotype × site × depth	20	n.s
