# Supplementary material for: High yielding biomass genotypes of willow (Salix spp.) show differences in below ground biomass allocation
Source: Biomass Bioenergy. 2015 Sep;80:114–27. doi: 10.1016/j.biombioe.2015.04.020 (PMC4547486; doi:10.1016/j.biombioe.2015.04.020)
Supplement: Supplementary file 1 [file mmc1.docx]

| **Destructive sampling date** | **Phenological stage** |
| --- | --- |
| **ROTATION 1** |  |
| 14^th^ June 2010 | Rapid canopy expansion |
| 26^th^ July 2010 | Canopy maximum |
| 8^th^/15^th^ Sept2010 | Growth cessation |
| 18^th^ Oct 2010 | Senescence |
| 21^st^ /24^th^ Jan 2011 | Dormancy |
| 5^th^/7^th^ Mar 2011 | Bud flush |
| 6^th^ June 2011 | Rapid canopy expansion |
| 29^th^ July/1^st^ Aug 2011 | Canopy maximum |
| 9^th^/12^th^ Sept 2011 | Growth cessation |
| 19^th^ /17^th^ Oct 2011 | Senescence |
| 23^rd^/27^th^ Jan 2012 | Dormancy |
| **ROTATION 2** |  |
| 5^th^ May 2012 | Re-growth from cutback |
| 31^st^ Jan/ 4^th^ Feb 2013 | Dormancy |
| 19^th^ June/24^th^ June 2013 | (Fine root sampling) |
| 6^th^/13^th^ Jan 2014 | Dormancy |

**Table S1.** Dates of the destructive sampling over the two rotations with the corresponding phenological stages. When the destructive samplings at the two sites didn’t coincide both dates are displayed (Harpenden/Aberystwyth).

**
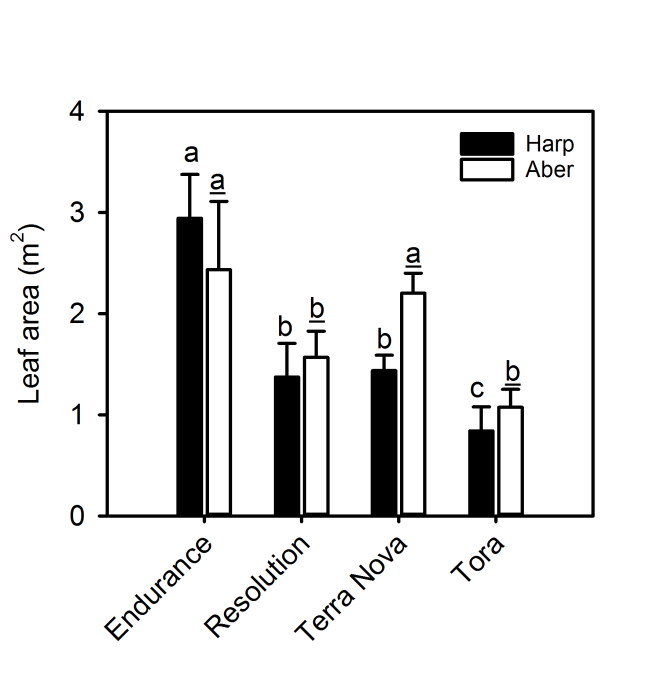
**

**Figure S1.** Leaf area (m^2^) for the four willow varieties grown at Harpenden (Harp) and Aberystwyth (Aber). Data are from the 6^th^ June 2011 destructive harvest and are means +SE of four replicates. Different letters indicate significant differences between the genotypes (*P*<0.05), with differences at Harpenden indicated by the lower case letters and differences at Aberystwyth the underlined lower case letters.


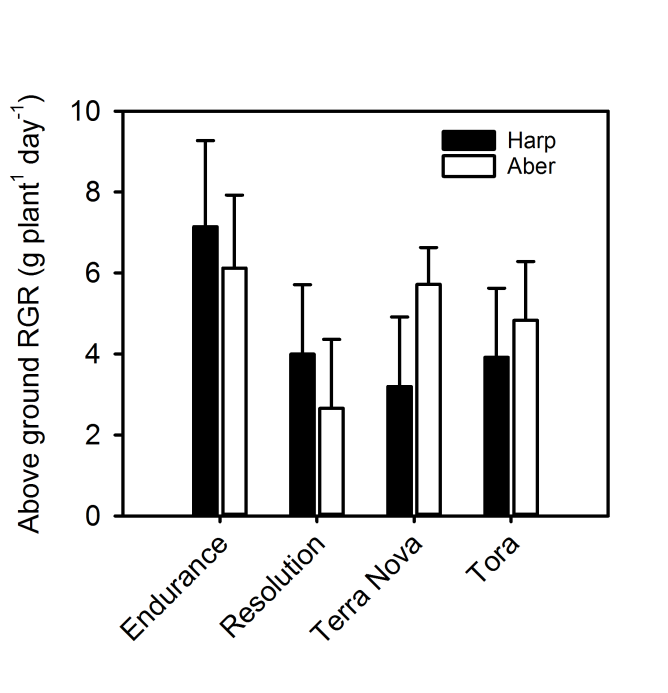


**Figure S2.** Relative growth rate (RGR, g plant^1^ day^-1^) for the four willow varieties grown at Harpenden (Harp) and Aberystwyth (Aber). RGR was calculated over the period between the 5/7^th^ March 2011 and June 6^th^ 2011 destructive harvest. Data are means +SE of four replicates.


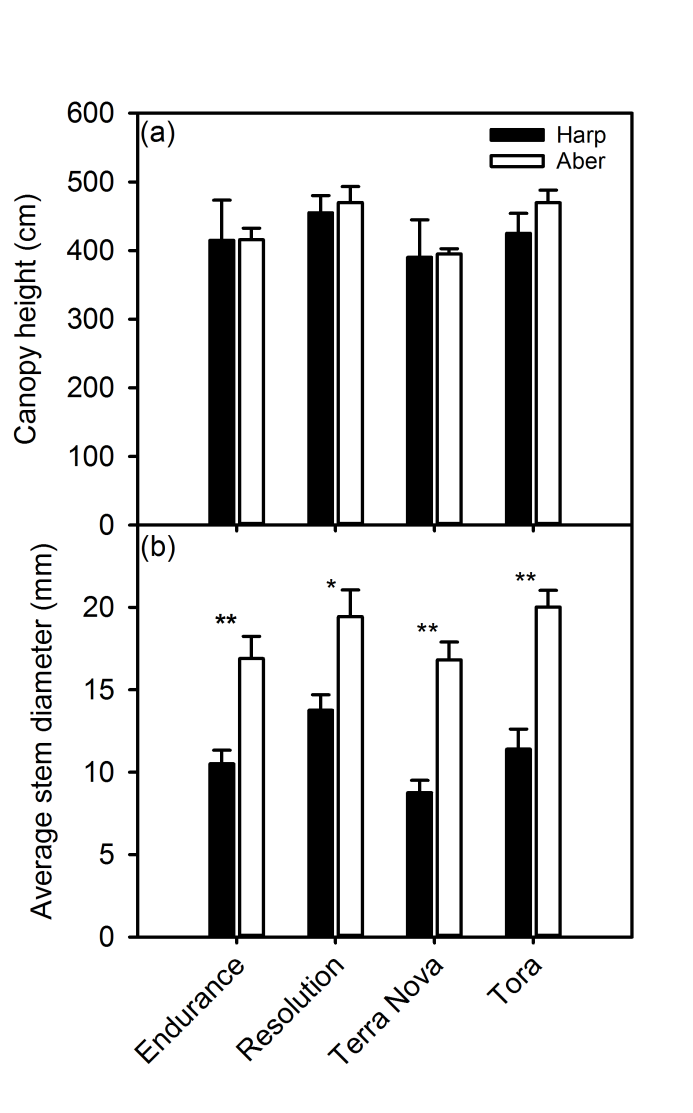


**Figure S3.** Canopy height (cm) and average stem diameter (mm) for the four willow varieties grown at Harpenden (Harp) and Aberystwyth (Aber). Data are from the January 2012 destructive harvest (end of the first rotation) and are means +SE of four replicates. Significant differences for the genotypes between the sites are indicated by **=<0.01 and *=<0.05.
